# Interleukin-1 Regulates Multiple Atherogenic Mechanisms in Response to Fat Feeding

**DOI:** 10.1371/journal.pone.0005073

**Published:** 2009-04-06

**Authors:** Janet Chamberlain, Sheila Francis, Zoe Brookes, Gary Shaw, Delyth Graham, Nicholas J. Alp, Steven Dower, David C. Crossman

**Affiliations:** 1 Cardiovascular Research Unit, School of Medicine and Biomedical Sciences, University of Sheffield, Sheffield, United Kingdom; 2 BHF Glasgow Cardiovascular Research Centre, University of Glasgow, Glasgow, United Kingdom; 3 Department of Cardiovascular Medicine, University of Oxford, John Radcliffe Hospital, Oxford, United Kingdom; 4 Functional Genomics, School of Medicine and Biomedical Sciences, University of Sheffield, Sheffield, United Kingdom; Department of Medicine, University of Hong Kong, China

## Abstract

**Background:**

Atherosclerosis is an inflammatory process that develops in individuals with known risk factors that include hypertension and hyperlipidaemia, influenced by diet. However, the interplay between diet, inflammatory mechanisms and vascular risk factors requires further research. We hypothesised that interleukin-1 (IL-1) signaling in the vessel wall would raise arterial blood pressure and promote atheroma.

**Methodology/Principal Findings:**

*Apoe^−/−^* and *Apoe^−/−^/IL-1R1^−/−^* mice were fed high fat diets for 8 weeks, and their blood pressure and atherosclerosis development measured. *Apoe^−/−^/IL-R1^−/−^* mice had a reduced blood pressure and significantly less atheroma than *Apoe^−/−^* mice. Selective loss of IL-1 signaling in the vessel wall by bone marrow transplantation also reduced plaque burden (p<0.05). This was associated with an IL-1 mediated loss of endothelium-dependent relaxation and an increase in vessel wall Nox 4. Inhibition of IL-1 restored endothelium-dependent vasodilatation and reduced levels of arterial oxidative stress.

**Conclusions/Significance:**

The IL-1 cytokine system links atherogenic environmental stimuli with arterial inflammation, oxidative stress, increased blood pressure and atherosclerosis. This is the first demonstration that inhibition of a single cytokine can block the rise in blood pressure in response to an environmental stimulus. IL-1 inhibition may have profound beneficial effects on atherogenesis in man.

## Introduction

Drugs to decrease plasma lipid levels and blood pressure are the basis of the medical treatment strategy for cardiovascular disease. Dietary modification of macronutrient and salt intakes has also been studied with some large effects upon systolic blood pressure in particular [1]–[3]. However, the exact interplay between the well-described plaque-based inflammatory processes of atherogenesis [2], [4] and the conventional risk factors of lipids and blood pressure remains poorly understood. We hypothesized that the pro-inflammatory cytokine interleukin-1 (IL-1), known to have an association with atherosclerosis, is the link between vascular responses and high fat feeding.

In the arterial wall, IL-1 is secreted primarily by monocytes and macrophages but also by endothelial and smooth muscle cells [5], [6]. IL-1 is an apical cytokine initiating inflammatory signals from bacterial products, chemical injury, complement activation and clotting factors. There are two agonistic cytokines, IL-1α and β which both signal via the type-I IL-1 receptor (IL-1R1). The system is tightly regulated by a natural antagonist, interleukin-1 receptor antagonist (IL-1ra) which also binds to IL-1R1 but does not produce a signal, and a non-signaling cell surface receptor, IL-1 receptor type-II, which acts as a decoy at the cell membrane or when shed as a soluble circulating decoy receptor [7], [8].

IL-1 is a powerful inflammatory stimulus to endothelial and vascular smooth muscle cells with effects that are plausibly linked with known mechanisms of atherogenesis [9]. Levels of IL-1 are increased in coronary arteries affected by atherosclerosis [10], [11], and inhibition of IL-1 in animal models is associated with reduced amounts of atheroma [12]–[16] as well as neointima following angioplasty [17]. Excess IL-1 leads to vascular cell oxidative stress, which is linked with elevation of arterial blood pressure mediated by adverse effects of oxidative stress upon bioavailable nitric oxide.

We report here the cardiovascular effects of abolishing IL-1 signaling either by genetic deletion of IL-1R1 or by administration of IL-1ra in proatherogenic *Apoe^−/−^* mice fed atherogenic diets. We also show a link between fat feeding and blood pressure that is mediated via IL-1 through modulation of the NADPH-oxidase subunit 4 (Nox 4).

## Materials and Methods

More detailed methods can be found in the online supplemental material (Text S1).

### Animals

*Apoe^−/−^/IL-R1^−/−^* mice were generated at JAX labs by cross breeding of *Apoe^−/−^* (JAX 2052) with *IL-R1^−/−^* (JAX 3245) mice. All mice were on a C57BL/6 background. Male mice, 8 weeks of age, (12 per group) were fed normal chow (4.3% fat, 0.02% cholesterol), Western diet (21% fat, 0.15% cholesterol, 0.03% cholate, 0.296% sodium) or Western High Cholate (WHC or Paigen) diet (18.5% fat, 0.9% cholesterol, 0.5% cholate, 0.259% sodium) for 8 weeks. No differences in body weights between the strains were observed either with or without fat feeding. Diets were supplied by Special Diet Services, UK. All animal experiments were approved by the University of Sheffield Project Review Committee and conformed to UK Home Office ethical guidelines.

### Quantification of Atherosclerotic Lesions

Histologically stained paraffin wax embedded sections were analysed as previously described [18].

### Blood Pressure Analysis

Systolic and diastolic blood pressures of mice were measured using a Visitech tail-cuff system (Visitech Systems, NJ, USA) and mean blood pressure calculated (supplementary text S1).

### Arteriolar Myogenic Reactivity and Responsiveness to Nitric Oxide

A pressure myograph system (Living Systems Instrumentation, USA) was used to determine arteriolar myogenic reactivity and responsiveness to nitric oxide, as previously described [19], [20].

### Bone Marrow transplantation

Irradiation and bone marrow transplantation of mice was performed as previously described [18] (text S1).

### Detection of Reactive Oxygen Species (ROS), NOS and Nitric Oxide

Superoxide production in mouse aortic tissue sections was detected *in-situ* [21]. Levels of ROS in aortic tissue were determined by chemiluminescent assay [22]. Nitric oxide synthase (NOS) enzymatic activity, and indirectly nitric oxide (NO) synthesis, was measured by the conversion of 14C L-arginine to 14C L-citrulline [23]. These methods are given in detail in the supplementary data. NO bioactivity in mice fed WHC was determined using an enzyme immunoassay for cyclic guanosine monophosphate (cGMP) (Cayman Chemical, UK).

### Quantitative Real time polymerase chain reaction

Quantification of Nox 2 and Nox 4 was performed by amplification of artery cDNA using a MX3000P instrument (Stratagene) with SYBR green dye, and normalization to 18S rRNA. For cultured cells, normalization was to β-actin. Optimized amplification conditions were 150 mmol/L primers, 2.5 mmol/L MgCl_2_, annealing at 60°C and extension at 72°C. Primer sequences were as described in [24].

### Statistical Analysis

Data groups were analyzed by t-test or one-way ANOVA followed by a Bonferroni multiple comparison post-test, as appropriate. Blood pressure measurements were analyzed by global non-linear regression, followed by an F-test. Vessel diameters were analyzed by two-way repeated-measures ANOVA. pD_2_, E_max_, L-NAME effects and wall: lumen ratios were compared by t-tests. Data are presented as mean±SEM. p<0.05 was regarded as a significant difference.

## Results

Further results can be found in the online supplementary text (Results S1).

### Effects of genetic deletion of IL-1R1 and diet type on atherosclerosis lesion development

**Figure 1:**
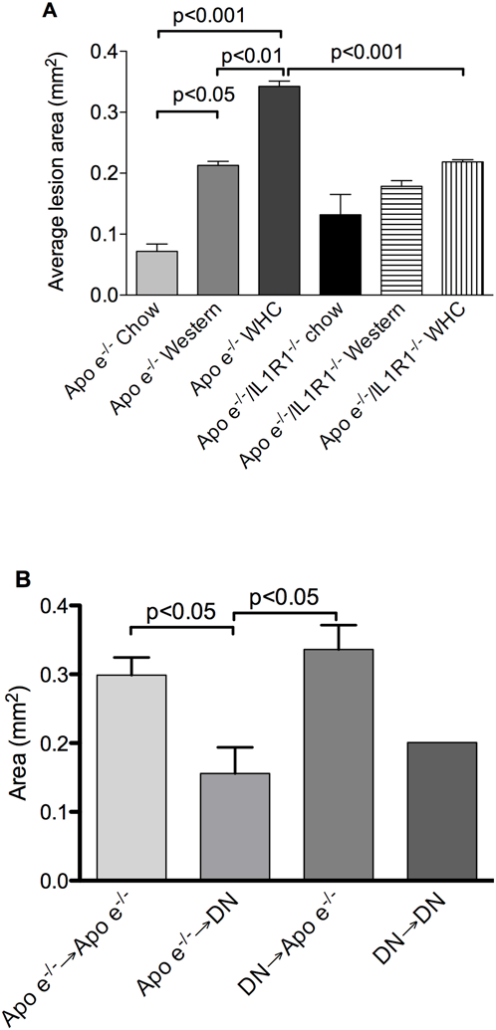
Atherosclerosis formation in mice following feeding of a high fat diet. Atherosclerotic lesion area in the aortic sinus of *Apoe^−/−^* and *Apoe^−/−^/IL-R1^−/−^* mice (a) and chimeric mice (b) fed a high fat and chow diets. Apoe→DN denotes *Apoe^−/−^/*IL-1R1^−/−^ (DN) mice receiving *Apoe^−/−^* bone marrow (n = 3); DN→Apoe denotes *Apoe^−/−^* mice receiving *Apoe^−/−^/*IL-1R1^−/−^ marrow (n = 9).

As expected, *Apoe^−/−^* mice fed high fat diets displayed significant increases in atherosclerotic lesion area compared to mice fed chow, with the Western diet with high cholate (WHC) causing the a greatest increase (Figure 1a). In contrast, lesions developed by *Apoe^−/−^/IL-R1^−/−^* mice fed these high fat diets were not significantly different to *Apoe^−/−^/IL-R1^−/−^* mice fed on chow. Analysis of each different diet type separately revealed no significant difference in lesion area of *Apoe^−/−^* and *Apoe^−/−^/IL-R1^−/−^* mice fed chow (p = 0.3), or Western diet (p = 0.4). However, when fed the WHC diet, *Apoe^−/−^/IL-R1^−/−^* mice developed a 36% smaller atherosclerotic lesion in the aortic sinus compared to *Apoe^−/−^* mice (p<0.001) (Figure 1a). Representative images of the microscopic appearance of the lesions are shown in Figure S1. Characterisation of the lesions revealed no overt differences with respect to strain or diet (Results S1, table S1, Table S2, figure S2). Body weight did not differ between mice of any group (data not shown).

### Atherosclerotic lesion formation in IL-1R1 chimeric mice fed a WHC diet

*Apoe^−/−^* and *Apoe^−/−^/IL-R1^−/−^* chimeric mice were used to determine the relative contribution of IL-1 signaling in cells derived from the bone marrow and non-bone marrow (tissue cells) upon the development of atherosclerosis following fat feeding. Irradiated *Apoe^−/−^* mice receiving *Apoe^−/−^* bone marrow had large, and *Apoe^−/−^/IL-R1^−/−^* mice receiving *Apoe^−/−^/IL-R1^−/−^* marrow relatively little, atherosclerosis in their aortic sinus following 8 weeks on a WHC diet, as expected and analogous to *Apoe^−/−^* and *Apoe^−/−^/IL-R1^−/−^* non-chimeric animals.

Interestingly, *Apoe^−/−^* animals receiving *Apoe^−/−^/IL-R1^−/−^* bone marrow (i.e. chimera is capable of IL-1 signaling in vessel wall/tissue cells) did not develop a lesion that was significantly different from *Apoe^−/^* mice receiving *Apoe^−/−^* marrow (Figure 1b). In contrast, *Apoe^−/−^/IL-R1^−/−^* mice receiving *Apoe^−/−^* bone marrow (i.e. chimera is not able to signal via IL-1 in vessel wall/tissue cells) developed a 1.9-fold smaller lesion than the *Apoe^−/−^* to *Apoe^−/−^* controls (p<0.05). The lesion size between *Apoe^−/−^/IL-R1^−/−^* into *Apoe^−/−^* and *Apoe^−/−^* into *Apoe^−/−^/IL-R1^−/−^* mice also differed significantly (p<0.05) (Figure 1b).

Pathological analysis, by chromosome painting, of these animals showed all had engrafted donor marrow (figure S3).

### Blood pressure responses to fat feeding

**Figure 2:**
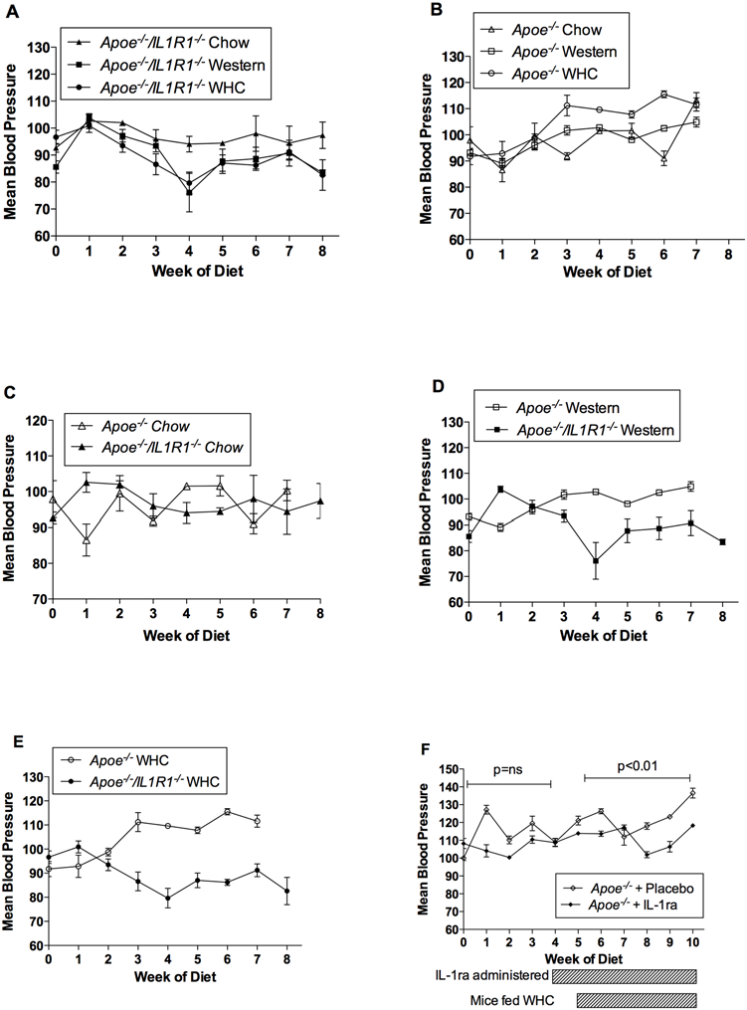
Blood pressure of mice following feeding of a high fat diet. Mean blood pressure in *Apoe^−/−^/IL-1R1^−/−^* (a) and *Apoe^−/^* (b) mice fed all 3 diets, and *Apoe^−/−^/IL-1R1^−/−^* compared to *Apoe^−/−^* mice fed chow (c) Western (d) and WHC (e). (f) Mean blood pressure in *Apoe^−/−^* mice administered with hrIL-1ra or placebo, fed WHC diet. n = 4 for all groups.

There was no difference in mean blood pressure in *Apoe^−/−^/IL-R1^−/−^* mice fed the Western, WHC or chow diet (figure 2a). However, *Apoe^−/−^* mice fed high fat diets had increased blood pressure compared with chow fed animals, with WHC-fed mice having a greater elevation than Western fed animals (p<0.05 WHC vs Western or chow)(figure 2b).

When comparing mouse strains, no difference was seen in mean blood pressure between *Apoe^−/−^/IL-R1^−/−^* or *Apoe^−/−^* fed a normal chow diet over an 8-week period (p = ns)(figure 2c). However, on feeding high fat diets, *Apoe^−/−^/IL-R1^−/−^* mice fed either the Western or WHC diet had a significantly lower blood pressure (a level comparable to *Apoe^−/−^* mice on chow diet) than the *Apoe^−/−^* mice fed the equivalent diet (p<0.001; p<0.0001 respectively, figure 2d, 2e).

*Apoe^−/−^* mice on WHC diets infused with IL-1ra using an osmotic pump also had a significantly lower blood pressure than *Apoe^−/−^* mice given placebo (Figure 2f). Elevations of IL-1ra in these animals was confirmed by ELISA, and found to be 1435.1+/−118.7 pg/ml compared to 0.215+/−0.1452 pg/ml in placebo mice.

### Effect of IL-1R1 deletion on vascular reactivity and compliance in mice fed WHC diet

**Figure 3:**
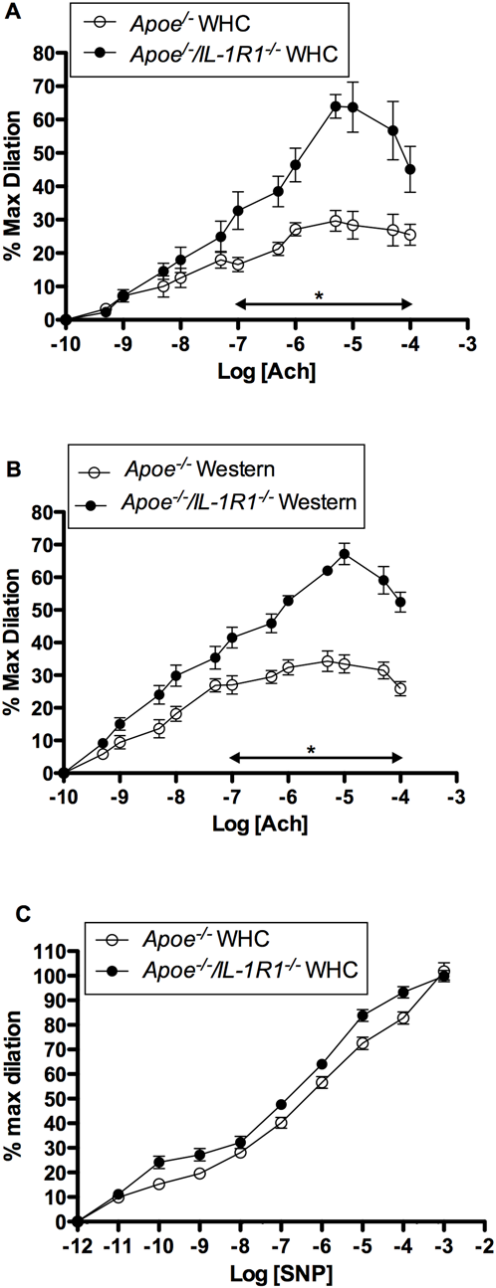
Vascular reactivity of arterioles from *Apoe^−/−^* and *Apoe^−/−^/IL-R1^−/−^* mice. (a–b) % maximum dilation of pre-constricted mesenteric arterioles (EC_80_PE) to acetylcholine. (c) Endothelium-independent relaxation responses assessed by superfusion of sodium nitroprusside (SNP) were not different between *Apoe^−/−^* and *Apoe^−/−^/IL-R1^−/−^* mice both fed a WHC diet (n = 6 per group).

Mesenteric arteries from *Apoe^−/−^* and *Apoe^−/−^/IL-R1^−/−^* mice fed Western and WHC diets did not differ in their sensitivity to phenylephrine (PE) (table S3). However, preconstricted arteries from *Apoe^−/−^/IL-R1^−/−^* mice fed these diets displayed significantly greater vasodilator sensitivity to acetyl choline (ACh) (p<0.05; Figures 3a and b) compared to *Apoe^−/−^* animals. The vasodilatory response to ACh was also significantly different between chow, Western and WHC diets in *Apoe^−/−^* mice (p<0.05). In *Apoe^−/−^/IL-R1^−/−^* mice fed WHC diet there was greater constriction (19.1+/−1.3%) compared to *Apoe^−/−^* (8.6+/−2.7%) following incubation with N(G)-nitro-L-arginine methyl ester (L-NAME) (p = 0.003), without a significant difference between pD_2_ (negative logarithm of EC_50_: concentration required for half maximum response) (table S3).

Both *Apoe^−/−^* and *Apoe^−/−^/IL-R1^−/−^* mice demonstrated similar internal luminal diameters of small mesenteric arteries increased in response to increasing intraluminal pressure (active response), with *Apoe^−/−^* mice fed either diet showing a trend towards a reduced myogenic response (figure S4a, S4b). However, only *Apoe^−/−^/IL-R1^−/−^* mice fed WHC diet demonstrated a greater active pressure-diameter response compared to chow fed *Apoe^−/−^* mice (figure S4a).

There was no difference in the wall-to-lumen ratio (vascular smooth muscle thickness/external diameter at 80 mmHg; *h*/R) [19] between the two groups (0.115+/−0.007 *Apoe^−/−^/IL-R1^−/−^* vs 0.111+/−0.011 *Apoe^−/−^*, n = 6). Endothelium-independent relaxation responses, assessed by superfusion of sodium nitroprusside (SNP), also did not differ (figure 3c).

### Effect of IL-1R1 deletion on NO activity, ROS, NOS and NOX expression in mice fed WHC diet

The induction of Nox4 mRNA and generation of ROS in response to IL-1 stimulation was confirmed in arteriolar endothelial cells (Figure S5). ROS were not generated in response to IL-1 in endothelial cells isolated from *Apoe^−/−^/IL-R1^−/−^* mice (figure S5). In contrast, Nox 4 mRNA was reduced in IL-1 stimulated vascular smooth muscle cells (VSMC) (figure S6) as previously shown [25].

The vessel wall mechanisms that could contribute to the observed vascular reactivity effects were investigated by examining the expression of NO, ROS, NOS and Nox isoforms in WHC diet-fed animals, as this diet gave the largest difference in blood pressure and atheroma between animals with and without the ability to signal via IL-1R1.

**Figure 4:**
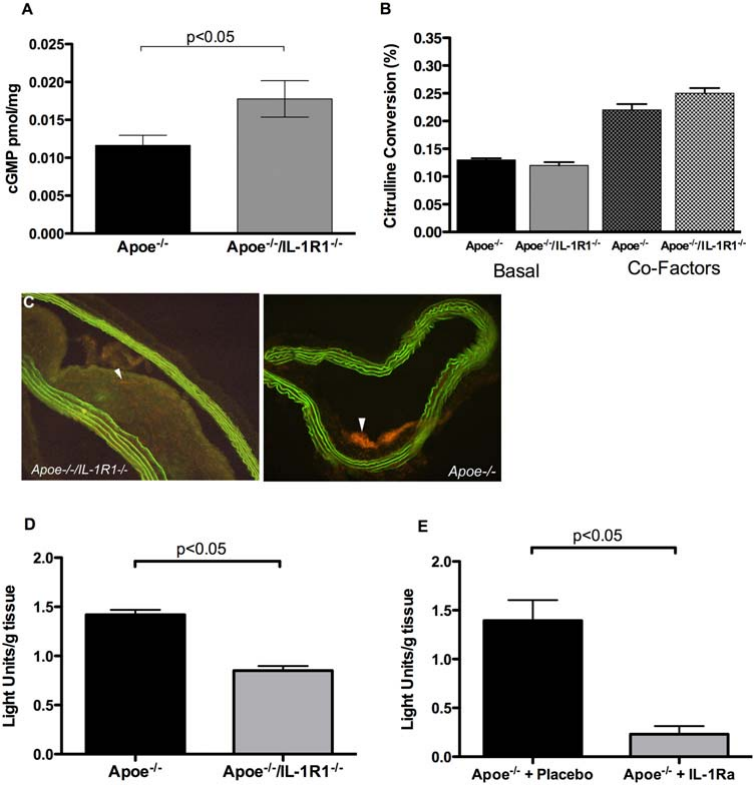
NO activity, ROS and NOS expression in mice fed WHC diet. (a) cGMP in lung homogenates (n = 14), (b) eNOS enzymatic activity (n = 6) and (c) *in-situ* ROS (white arrowhead) in *Apoe^−/−^/IL-R1^−/−^* and *Apoe^−/−^* mice fed WHC. *(Note: images chosen illustrate ROS levels not atherosclerotic lesion size)*. Chemiluminescent quantification of ROS in mice fed WHC (d) and *Apoe^−/−^* mice fed WHC diet (n = 9) and administered hrIL-1ra (n = 4) (e).

NO bioactivity was increased in *Apoe^−/−^/IL-R1^−/−^* mice fed a WHC diet compared with WHC fed *Apoe^−/−^* mice (n = 9–11, p<0.05) (Figure 4a). Activity of eNOS in the presence or absence of eNOS co-factors did not differ between *Apoe^−/−^/IL-R1^−/−^* mice fed a WHC diet compared with *Apoe^−/−^* mice (p = ns) (Figure 4b). Qualitative *in situ* detection of ROS showed production in atherosclerotic plaques was reduced in *Apoe^−/−^/IL-R1^−/−^* compared to *Apoe^−/−^* mice fed WHC diet (Figure 4c). Quantitative measurement of aortic ROS production showed ROS to be significantly reduced in *Apoe^−/−^/IL-R1^−/−^* mice on a WHC diet (n = 9, p<0.05)(Figure 4d). ROS production was also suppressed in *Apoe^−/−^* mice on a WHC diet by a 4-week infusion of IL-1ra (n = 3, p<0.05)(Figure 4e). Generation of ROS in response to feeding a high fat diet was also confirmed (figure S7). *Apoe^−/−^* mice fed WHC had significantly more ROS than those fed chow alone, with Western diet giving intermediate levels of ROS. ROS levels were also significantly higher in *Apoe^−/−^/IL-R1^−/−^* mice fed WHC compared to chow (0.85+/−0.14 compared to 0.51+/−0.30 LU/g tissue, n = 9, p<0/01).

**Figure 5:**
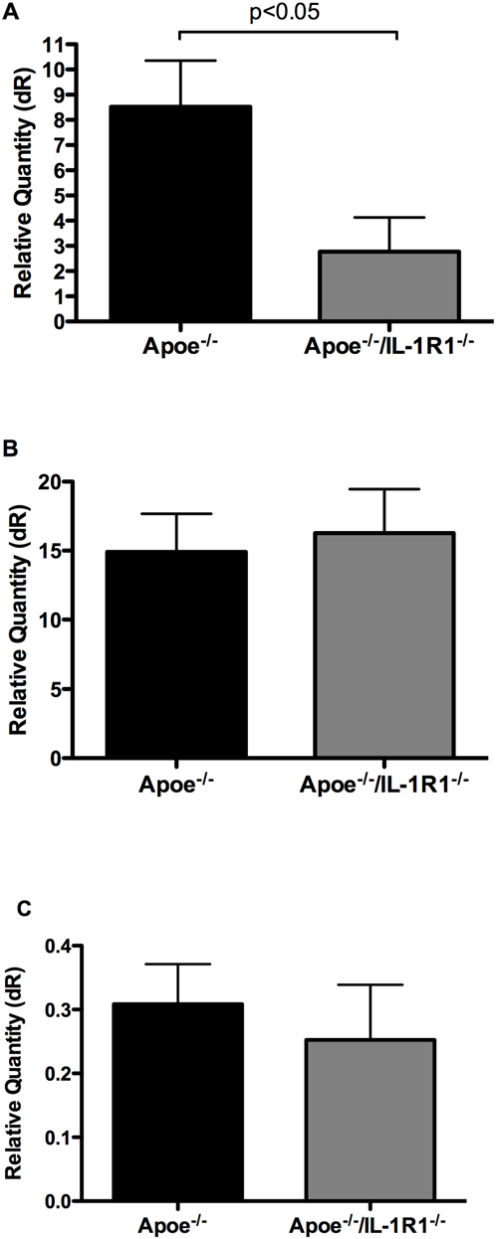
Nox expression following feeding of WHC diet. Expression of Nox is selectively reduced in *Apoe^−/−^/IL-R1^−/−^* mice fed WHC. (a) qRT-PCR shows reduced (p<0.05) levels of Nox 4 in the vascular wall of *Apoe^−/−^/IL-R1^−/−^* mice fed WHC diet. Levels of Nox 2 (b) and Nox 1 (c) do not differ. n = 12 per group.

Nox 4 expression in the whole arterial wall was significantly reduced in *Apoe^−/−^/IL-R1^−/−^* mice fed the WHC diet compared to *Apoe^−/−^* mice (Figure 5a). Expression of Nox 1 and 2 however, did not differ between these two mouse strains on this diet (figure 5b, 5c).

## Discussion

The *Apoe^−/−^* mouse fed a high fat diet is an established model of atherogenesis. On a Western or a Western high cholate diet (WHC, sometimes called the Paigen diet), *Apoe^−/−^* mice develop aortic atheroma [26] and blood pressure is known to rise within 1 to 3 months of feeding. We show here that IL-1 inhibition reduces atheroma formation and prevents the associated rise in blood pressure in *Apoe^−/−^* mice fed high fat diets.

IL-1 inhibition was achieved by generating mice deficient for both *Apoe* and IL-1 receptor 1 (IL-1R1) genes. As expected, aortic atheroma was significantly reduced in *Apoe^−/−^/IL-R1^−/−^* mice fed WHC compared with *Apoe^−/−^* controls. This is in agreement with previous studies that showed decreased atherosclerosis in IL-1β^−/−^ and IL-1α^−/−^ mice fed a high cholate diet [27] and with data from a heterozygote *Apoe^−/−^* model [16]. In addition, both high fat diets were associated with increased markers of inflammation that were attenuated in *Apoe^−/−^/IL-1R1^−/−^* mice (Results S1). Interestingly, IL-1β and IL-1ra plasma levels in mice were not changed in response to fat-feeding, This was not unexpected: mature plasma IL-1β is a difficult parameter to interpret as a non-secreted protein and IL-1ra behaves as an acute phase reactant with a large variation in levels [28]. Any true changes in inflammatory markers are more likely to occur locally within the vessel wall itself.

*Apoe^−/−^* mice fed a high fat diet display a rise in blood pressure that is not seen in *Apoe^−/−^/IL-R1^−/−^* mice fed equivalent diets. The specificity of this was confirmed by abolition of the fat-feeding induced blood pressure rise in *Apoe^−/−^* mice given IL-1ra. Stroke-prone spontaneously hypertensive rats (SHRSP) showed no difference in blood pressure if given IL-1ra (data not shown), suggesting that IL-1ra does not act as a non-specific vasodilator.

To study the mechanism of the attenuation of the blood pressure rise in the *Apoe^−/−^/IL-R1^−/−^* mice we investigated active arteriolar pressure-diameter responses and basal and agonist-induced NO production in *Apoe^−/−^* and *Apoe^−/−^/IL-R1^−/−^* fat-fed mice. The greater vessel diameter in response to increasing pressure in vessels from *Apoe^−/−^/ILR1^−/−^*, together with no observed difference in sensitivity to PE between groups or in vascular smooth muscle constriction for the given degree of muscle shortening (wall: lumen ratio), as well as the preserved response to SNP, all suggest a specific endothelial response.

The strikingly preserved agonist-induced NO dependent vasodilatation of resistance vessels from *Apoe^−/−^/IL-1R1^−/−^* mice compared to *Apoe^−/−^* in both diet groups as well as the greater contraction in response to L-NAME in the fat-fed *Apoe^−/−^/IL-1R1^−/−^* mice indicate a fundamental increase in bioavailable vessel wall nitric oxide as a result of selective loss of IL-1 signaling. This is emphasised by the observed increase in basal cGMP production seen in these mice. The activity of eNOS, in the presence or absence of eNOS co-factors, did not differ between the two mouse strains suggesting that the difference in bioavailable NO attributable to selective IL-1 inhibition is mediated by increased consumption of NO via increased arterial oxidative stress.

In support of these data, there was consistent reduction of arterial ROS levels in mice unable to signal via IL-1. Investigation of Nox isoforms, known to regulate NADPH activity [29], [30], indicated a Nox-4 selective rise in response to fat feeding in the *Apoe* ^−/−^ mouse attenuated by abolition of IL-1 signaling. Taken together, these results suggest a central role for IL-1 in modulating the blood pressure response to high lipids and that this is via a selective reduction in bioavailable nitric oxide.

The chimeric mouse experiments were conducted to examine the compartment in which IL-1 signaling was important. Only in chimeras where tissue cells were unable to signal via IL-1 was a there a decrease in atherosclerotic lesion size. These data complement a recent study by Kamari *et al.* who showed reduced atherosclerosis in wild type, WHC-fed mice reconstituted with either IL-1β^−/−^ or IL-1α^−/−^ bone marrow [27].

We also demonstrate that vessel wall ROS production and Nox 4 levels are altered between the non-chimeric *Apoe^−/−^* and *Apoe^−/−^/IL-1R1^−/−^* strains on high fat diets. The myography experiments also suggest a selective endothelial loss of function. Thus, the data presented here add to the hypothesis that IL-1 signaling within the blood vessel wall cells is the mechanism involved in the blood pressure and atherogenic responses to fat feeding in *Apoe^−/−^* mice.

Our studies examined the effects of two different pro-atherogenic diets, administered for 8 weeks. Of these diets, the WHC diet is known to be the most aggressive, giving a chronic inflammatory response compared to the acute response of a standard Western (high cholesterol/low cholate) diet [31]. Besides being a normal component of bile in vertebrates, cholate (E1000) is an emulsifier and foam stabilizing agent used in food manufacturing which leads to very high circulating cholesterol concentrations in plasma. Given the difference in plasma cholesterol load, it is interesting that the poor vascular reactivities of vessels from mice fed both diets were similar with a clear gain of function seen in mice lacking the ability to signal via IL-1R1, irrespective of the cholate content. Atheroma development and blood pressure elevation were graded in magnitude through chow to the Western and WHC diets, with the WHC having the greatest effect. In keeping with this, the greatest level of attenuation of biological events by IL-1 inhibition was seen in the WHC diet fed animals. These all suggest a specificity of IL-1 in the mediation of fat fed vascular events.

The WHC diet can cause intense metabolic disruption, hepatic dysfunction and inflammatory activation. We did not see these effects. Additionally, we saw similar index data (e.g. blood pressure and atheroma formation) for mice fed on the Western and the WHC diet, though the magnitude of the blood pressure effects was reduced with the Western diet. We feel, therefore, that where we have used the WHC diet for further detailed experimentation that this is justified and that the results derived are the result of elevation in cholesterol: the Western diet giving intermediate cholesterol levels and intermediate phenotype, and the WHC diet giving rise to high cholesterol and an advanced phenotype.

There is considerable interest in harnessing knowledge of basic inflammatory mechanisms of atherogenesis for therapeutic benefit. We show that dietary manipulation in a murine model of atherosclerosis is associated with a rise in blood pressure, an effect attenuated by selective inhibition of a single cytokine. High fat diets may be particularly linked with elevation in blood pressure [3], [32]. It is clear that in humans, dietary alterations away from a standard Western diet to those lower in fat, but which are isocaloric and balanced for sodium content, or which are supplemented with omega-3 fatty acids, also lower blood pressure [1], [33]–[35]. Omega-3 fatty acids have also been shown to reduce cyclooxygenase-2 and NADPH oxidase activity [36], both of which are induced by IL-1. This suggests that the link between diet and blood pressure may be through the IL-1 cytokine system, and may indicate a role for IL-1 inhibition in the control of hypertension in man.

In our experiments, there was a concomitant reduction in blood pressure and atheroma development. In fat-fed *Apoe^−/−^* mice, development of atheroma is not driven directly by the rise in blood pressure [37]. It seems likely therefore, that the IL-1 mediated increase in ROS and decreased NO production are the central mechanism of atheroma development as both of these have individually been implicated in atherogenesis. The absence of difference in lipid levels between the different mouse strains on the same diets (results S1) confirms the likely effect of IL-1 inhibition on atherogenesis being a ROS/NO-based mechanism.

These data raise the possibility of an entirely novel strategy for prevention of atherosclerosis. An anti-IL-1 based approach would be expected to have a beneficial effect upon atherogenic mechanisms at multiple levels. Indeed, very recent data have indicated a beneficial effect of IL-1ra upon patients with type II diabetes [38], ventricular function [39] and myocardial infarction in mice [40] and a similar approach might allow mitigation of the atherogenic potential of Western diets. We anticipate that targeted pharmacological intervention to inhibit IL-1 in man will be beneficial in preventing atheroma development and the clinical consequences that arise from this important disease of developed nations.

## Supporting Information

Text S1 Expanded methods.
(0.04 MB DOC)

Results S1 Additional data
(0.04 MB DOC)

Figure S1 Microscopic appearances around the aortic sinus of ApoE^−/−^ mice fed chow, Western, and WHC compared with ApoE^−/−^/IL-1R1^−/−^ mice. Original magnification×2.
(1.18 MB TIF)

Figure S2 Modulation of IL-1 signaling decreases acute phase reactant serum amyloid A (SAA) levels. SAA was elevated in Apoe^−/−^ mice on both high fat diets, an increase that was significantly reduced in the Apoe^−/−^/IL-R1^−/−^ mice on equivalent diets. (n = 9–20)
(4.80 MB TIF)

Figure S3 Chromosome painting of bone-marrow transplanted mice. No Y-chromosomes were seen in male mice transplanted with female bone marrow, confirming engraftment was successful. Male recipients of male bone marrow all have Y-chromosomes, as expected, as a positive control for this method.
(2.45 MB TIF)

Figure S4 Vascular reactivity of arterioles from Apoe^−/−^ and Apoe^−/−^/IL-R1^−/−^ mice. Intraluminal arteriolar diameter in response to increasing pressure (0–120 mmHg). *P<0.05 Apoe^−/−^/IL-R1^−/−^ (n = 6) and **P<0.05 Apoe^−/−^ WHC (n = 6) versus Apoe^−/−^ chow (n = 4).
(0.61 MB TIF)

Figure S5 ROS and Nox 4 expression in endothelial cells in culture. Increased ROS are seen following IL-1β stimulation of endothelial cells isolated from Apoe^−/−^ mice (n = 6) (a) and human coronary endothelial cells (hCAEC) (n = 6) (b). However, no increase in ROS are seen in ECs isolated from Apoe^−/−^/IL-1R1^−/−^ mice (n = 6) (a). Nox 4 mRNA expression is increased in hCAEC stimulated with IL-1β (n = 5) (c).
(9.90 MB TIF)

Figure S6 ROS and Nox 4 expression in vascular smooth muscle cells in culture. No significant difference in ROS are seen following IL-1β stimulation of VSMCs (n = 6) (a). Nox 4 mRNA expression is decreased in VSMC stimulated with IL-1β (n = 1) (b).
(0.30 MB TIF)

Figure S7 ROS generation in Apoe^−/−^ mice following feeding of a high fat diet. Mice fed WHC had significantly more ROS than those fed chow alone, with Western diet giving intermediate levels of ROS. (n = 9).
(2.79 MB TIF)

Table S1 Lipid, glucose and ALT levels in ApoE^−/−^/IL-1R1^−/−^ and ApoE^−/−^ mice fed chow, Western High Cholate (WHC), and Western diets. Data represents mean+/−SEM.
(0.05 MB DOC)

Table S2 Plasma levels of IL-1ra, IL-1β, IL-1α and IL-6 in ApoE^−/−^/IL-1R1^−/−^ and ApoE^−/−^ mice fed chow, Western high cholate (WHC), and Western diets, determined by ELISA. Data represents mean+/−SEM.
(0.04 MB DOC)

Table S3 Potency and efficacy of vasoconstrictor response to phenylephrine in pressurised mesenteric arterioles from Apoe^−/−^/IL1R1^−/−^ and Apoe^−/−^ mice fed a Western high cholate (WHC), Western, or Apoe^−/−^ mice fed chow diet, assessed by pD2 (negative logarithm of EC50: concentration required for half maximum response) and Emax (percentage maximum constriction). All data are means+SEM. p = ns.
(0.04 MB DOC)
