# Supplementary material for: Interleukin-1 Regulates Multiple Atherogenic Mechanisms in Response to Fat Feeding
Source: PLoS One. 2009 Apr 6;4(4):e5073. doi: 10.1371/journal.pone.0005073 (PMC2661361; doi:10.1371/journal.pone.0005073)
Supplement: Text S1 — Expanded methods. (0.04 MB DOC) [file pone.0005073.s001.doc]

**Expanded materials & methods:**

***Blood Pressure Analysis of mice.***

Systolic and diastolic blood pressure of mice was measured using a Visitech tail-cuff system (Visitech Systems, NJ, USA) and mean blood pressure calculated.

To ensure stress levels of mice were kept to a minimum, a single handler was used throughout the experiment and mice were subjected to one week of training (blood pressure and pulse readings were taken, but the data discarded) prior to starting analysis. Measurements were taken at the same time, daily to avoid normal daily variance in blood pressure. In addition, the blood pressure was taken on the same part of the tail every day.

During analysis, 10 measurements were taken each day, and mean blood pressure and standard deviation calculated for each ‘data day’ and week (total of 50 readings per mouse per week, 10 per day). On each day, individual data points were rejected if the blood pressure was below 40 or above 210 mmHg, or if it was outside of 2 standard deviations from the mean. All data for a day was rejected if there were less than 4 valid readings. Data for a week was rejected if it did not have at least 3 valid days of measurements.

One week of baseline readings on chow diet were taken for each mouse, prior to feeding of Western or WHC diets.

These data were analysed by global non-linear regression. This statistical test analyses an entire family of data sets simultaneously (rather than one set at a time), sharing one or more parameters between data sets. For each shared parameter, global non-linear regression finds one best-fit value that applies to all the data sets. In this case, blood pressure was determined under control (chow fed) and treated (diet-fed) conditions, for different mouse genotypes, and global non-linear regression determines whether the difference between each blood pressure curve is convincing. The test does not compare individual timepoints, but instead treats the data globally to produce a single p value per comparison.

## *Plasma lipid measurements*

After fasting for 9 hours, plasma total cholesterol levels, high density lipoprotein, triglyceride, glucose, and alanine aminotransferase (ALT) levels were measured on an automated analyzer, Beckmann LX20.

***Enzyme-linked Immunosorbent assay (ELISA)***

Plasma levels of IL-1α, IL-1ra, IL-6 and IL-1β were determined by sandwich ELISA following the manufacturers protocol. (R&D Systems, UK)

## *Immunohistochemistry*

Paraffin-embedded sections of aortic sinus and brachiocephalic artery were stained immunohistochemically for α-smooth muscle actin (Dako) to visualize smooth muscle cells and F4/80 (Abcam) to visualize macrophages. Standard immunohistochemical techniques were applied.

***Quantification of Atherosclerotic Lesions.***

The area of lesion, and percent area of collagen per lesion was assessed by morphometric measurement of histologically stained paraffin-embedded sections using a Lucia image analysis software system (Lucia G, Nikon, UK). Lesion cellularity was determined by counting of cell nuclei. The values reported represent a mean from 5 sections for each animal.

***Arteriolar Myogenic Reactivity and Responsiveness to Nitric Oxide.***

Small arterioles (200-300µm), 2-3 branches distal from the superior mesenteric artery, were mounted on cannulae within a pressure myograph system (Living Systems Instrumentation, USA) for measurement of internal vessel diameter (intraluminal), vascular smooth muscle wall thickness and wall:lumen ratio without neural input [1, 2].

Following stabilization, the active pressure-diameter response (myogenic reactivity) was determined [1, 2]. All experiments were then performed on vessels pressurized at 80mmHg. A diameter cumulative dose response curve to phenylephrine (PE) (10^-9^ - 10^-4^M) or sodium nitroprusside (SNP) (10^-12^ – 10^-2^M) was constructed, allowing vessels to be pre-constricted with the EC_80_ concentration of PE or SNP before generation of a cumulative dose response curve to acetylcholine (ACh) (10^-10^ - 10^-4^M). The effects of endogenous NO on resting diameter were then tested by measuring the change in diameter after incubation with 100µM L-NAME for 45 minutes. At the end of the study the EC_80_ PE was also added in the presence of L-NAME, followed by the EC_80_ ACh to ensure L-NAME had been effective.

***Bone Marrow transplantation.***

Irradiation and bone marrow transplantation of mice was performed as previously described. [3]. All mice were 6 weeks old at point of bone marrow transplantation. Mice received 11Gy irradiation prior to transplantation of 1-2x10^6^ bone marrow cells. Female donor marrow was transplanted into male recipient mice, to enable confirmation of engraftment. Mice were fed WHC diet 5 weeks after bone marrow transplantation, for a period of 8 weeks.

The IL-1R1 strain was particularly sensitive to irradiation [3] and subsequent feeding of high fat diets. The survival rate of transplanting *Apoe^-/-^/IL-R1^-/-^* bone marrow into *Apoe^-/-^/IL-R1^-/-^* animals was 50%, decreasing to 12.5% by feeding a WHC diet. Pathological analysis, by chromosome painting, of these animals showed all had engrafted donor marrow (figure S3). There was a normal internal structure and no evidence of sepsis or viral particles.

***Detection of Superoxide production in mouse aortic tissue sections.***

Superoxide production in mouse aortic tissue sections was detected *in-situ* using the fluorescent probe dihydroethidium. Cryosections of thoracic aorta were incubated with Krebs HEPES buffer (99mmol/L NaCl; 4.7mmol/L KCl; 1.2mmol/L MgSO_4_; 1.0mmol/L KH_2_PO_4_; 1.9mmol/L CaCl_2_; 25mmol/L NaHCO_3_; 11.1mmol/L glucose; 20mmol/L NaHEPES) for 30 minutes at 37°C, followed by 5 minutes incubation in a light-protected chamber at 37°C with 2 µmol/L dihydroethidium (DHE; Molecular Probes). Some sections were also incubated with 500 U/mL polyethylene glycol-conjugated superoxide dismutase (PEG-SOD) (Sigma) to demonstrate specificity of ethidium fluorescence for superoxide. Images were captured using a Lucia image analysis software system (Lucia G, Nikon, UK).

***Detection of Reactive Oxygen Species in aortic tissue.***

Levels of ROS in aortic tissue were determined by chemiluminescent assay. Two 2.5mm rings of mouse aorta were equilibrated in Krebs/HEPES buffer for 30 minutes at 37°C before being placed in 2ml Krebs/HEPES buffer containing 100μmol/L luminol. Luminescence was counted after 10 minutes. Counts were taken every minute for 10 minutes, and an average count calculated. The tissue was then removed, dried at 90°C for 24 hours, and weighed. Chemiluminescence was expressed as light units/g dry tissue.

***Detection of Reactive Oxygen Species in cells.***

Levels of ROS in cells were determined by chemiluminescent assay. Endothelial or VSMC (50, 000 cells per well) were plated into wells of a 24-well plate containing a thermanox plastic coverslip (Nunc). The cells were cultured for 2 days before stimulation with 10ng/ml IL-1β for 18 hours at 37°C, followed by equilibration in Krebs/HEPES buffer for 30 minutes at 37°C. The coverslip, containing cells, was then removed and placed in 500μl Krebs/HEPES buffer containing 100μmol/L luminol. Luminescence was counted immediately. Counts were taken every minute for 5 minutes, and an average count calculated.

***Detection of NOS and Nitric Oxide in tissue.***

NOS enzymatic activity, and indirectly NO synthesis, was measured by the conversion of 14C L-arginine to 14C L-citrulline. Freshly harvested aortas were opened longitudinally and incubated in 250μl Krebs-HEPES buffer, with 1μM Ca ionophore A23187 (Sigma-Aldrich) and 5μl of 14C-labelled L-arginine (Amersham Biosciences UK Ltd) for 90 minutes at 37°C and the solution was then collected. The endothelium of the aortas was then lysed by three cycles of freeze-thawing in 250μl of water, and added to the previous solution collection. Sixty microliters of 10% trichloroacetic acid was then added to remove protein from the samples, which were centrifuged, and the supernatant collected. Samples were analyzed on a 250mm × 4mm SCX300 cation-exchange column (Sigma-Aldrich) using a Beckman HPLC system consisting of a 128 dual pump, 171 continuous-flow liquid scintillation detector, and System Gold Software version 6.4 (Beckman, High Wycombe, United Kingdom). Running conditions for elution at 1ml per minute were as follows: buffer A, 1mM citric acid [pH 2.2]; buffer B, 150mM sodium citrate [pH 3.0]; gradient of 100% A for 12 minutes, 50% A and 50% B for 18 minutes, and 100% B for 2 minutes. The column was regenerated after each sample using 10mM citric acid for 10 minutes and 1mM citric acid for 17 minutes. Scintillant fluid was mixed after elution from the column, prior to passage through the detector, at 0.5ml per minute. Standards of 14C-labelled L-arginine and citrulline were used to determine elution times. Citrulline peaks were integrated and expressed as a proportion of total 14C counts for each sample.

**References for supplementary methods:**

1. Brookes ZL, Kaufman S (2003) Myogenic responses and compliance of mesenteric and splenic vasculature in the rat. *Am J Physiol Regul Integr Comp Physiol*. 284: R1604-R1610.

2. Andrew PS, Kaufman S (2003) Guanylyl cyclase mediates ANP-induced vasoconstriction of murine splenic vessels. *Am J Physiol Regul Integr Comp Physiol*. 284: R1567-R1571.

3. Chamberlain J, Evans D, Dower S, Crossman D, Francis S (2006) IL-1β and signaling of IL-1 in vascular wall and circulating cells modulates the extent of neointima formation after vascular injury in mice. *Am J Pathol* 168: 1396-1403.
