# Supplementary material for: Interleukin-1 Regulates Multiple Atherogenic Mechanisms in Response to Fat Feeding
Source: PLoS One. 2009 Apr 6;4(4):e5073. doi: 10.1371/journal.pone.0005073 (PMC2661361; doi:10.1371/journal.pone.0005073)
Supplement: Results S1 — Additional data (0.04 MB DOC) [file pone.0005073.s002.doc]

Supplementary Results

***Lesion phenotyping***

Characterization of the lesions formed by each mouse strain, on each diet, revealed no difference in the percentage collagen content in the aortic sinus of any group (data not shown). The overall cellularity, α-smooth-muscle actin content, and macrophage numbers in lesions formed between mouse strains and diet likewise, did not differ significantly (data not shown).

*Deletion of IL-1R1 does not affect plasma lipid, glucose and ALT levels.*

As expected, plasma lipid analysis revealed elevated cholesterol and HDL levels in all mice fed the WHC diet (table S1). However, the cholesterol:HDL ratio did not differ significantly between mouse strains or diet type, suggesting the effects seen were a result of the biological responses to similar cholesterol levels. Neither glucose or alanine aminotransferase (ALT) levels varied between mouse strain or diet.

*Effect of genetic deletion of IL-1R1 on inflammatory modulators.*

Serum amyloid A was elevated in *Apo e^-/-^* mice on both high fat diets, an increase that was significantly reduced in the *Apo e^-/-^/IL-R1^-/-^*mice on equivalent diets (Figure S2).

No significant difference in levels of IL-1ra, IL-1β or IL-1α between mouse strain or diet were seen (Table S2).

No consistent pattern was observed in plasma IL-6 levels. *Apo e^-/-^*mice fed the Western diet had significantly higher levels of IL-6 compared to *Apo e^-/-^/IL-R1^-/-^*mice fed the same diet (p<0.001), but there was no significant difference seen between mice fed the WHC diet (table S2)**.** WHC-fed *Apo e^-/-^/IL-R1^-/-^*mice had significantly higher IL-6 levels than those fed Western diet (p<0.05).

***Reactive Oxygen Species and Nox 4 expression in EC and VSMCs.***

ROS production by endothelial cells was significantly increased following stimulation with IL-1β, detected by chemiluminescence assay. This increase was seen in both human coronary artery endothelial cells (hCAEC) (p<0.05, n=6) and endothelial cells isolated from lungs of Apoe^-/-^ mice (p<0.05, n=6). However, no increase was seen in endothelial cells isolated from Apoe^-/-^/IL-1R1^-/-^ mice (figure S5). Stimulation of vascular smooth muscle cells with IL-1β had no effect on ROS expression (p=ns, Figure S6).

Nox 4 expression by cultured hCAEC was also significantly following incubation with IL-1β (p<0.01, n=3) (Figure S6). However, incubation of VSMC with IL-1β resulted in a decrease in Nox 4 expression, in agreement with previously published data [1].

**Reference for supplementary results:**

1. Ellmark SHM, Dusting GJ, Ng Tang Fui M, Guzzo-Pernell N, Drummond GR (2005) The contribution of Nox4 to NADPH oxidase activity in mouse vascular smooth muscle. *Cardiovascular Research* 65:495-504.
