# Supplementary figures and images for: Interleukin-1 Regulates Multiple Atherogenic Mechanisms in Response to Fat Feeding
Source: PLoS One. 2009 Apr 6;4(4):e5073. doi: 10.1371/journal.pone.0005073 (PMC2661361; doi:10.1371/journal.pone.0005073)

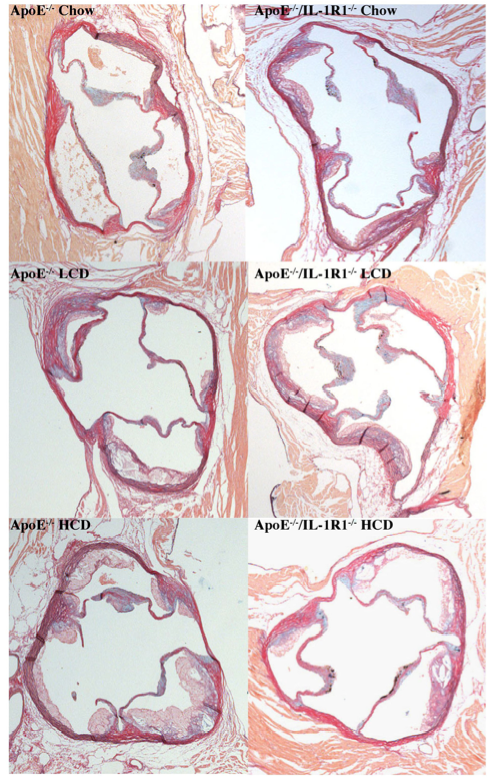

Supplement: Figure S1 — Microscopic appearances around the aortic sinus of ApoE−/− mice fed chow, Western, and WHC compared with ApoE−/−/IL-1R1−/− mice. Original magnification×2. (1.18 MB TIF) [file pone.0005073.s003.tif]

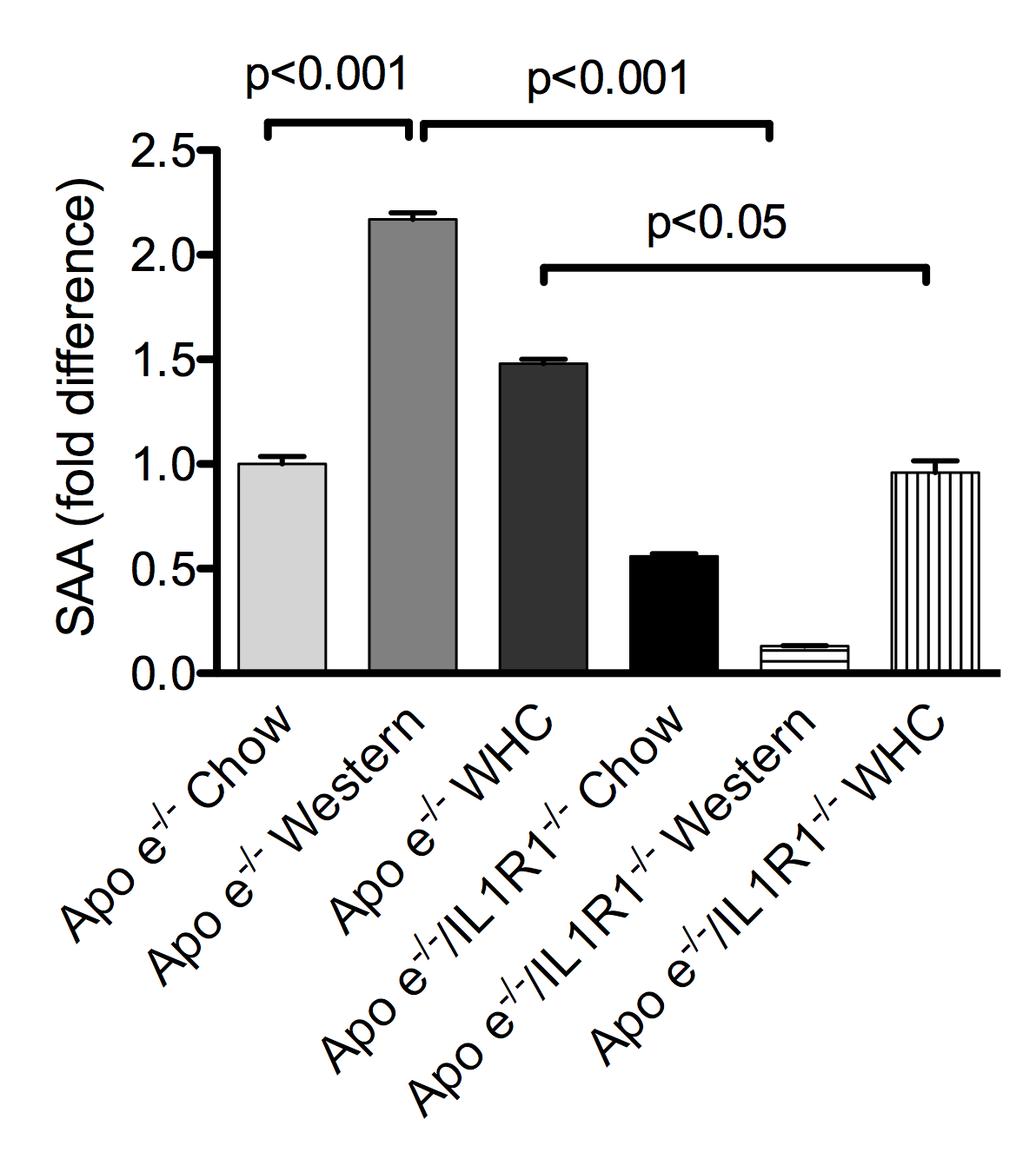

Supplement: Figure S2 — Modulation of IL-1 signaling decreases acute phase reactant serum amyloid A (SAA) levels. SAA was elevated in Apoe−/− mice on both high fat diets, an increase that was significantly reduced in the Apoe−/−/IL-R1−/− mice on equivalent diets. (n = 9–20) (4.80 MB TIF) [file pone.0005073.s004.tif]

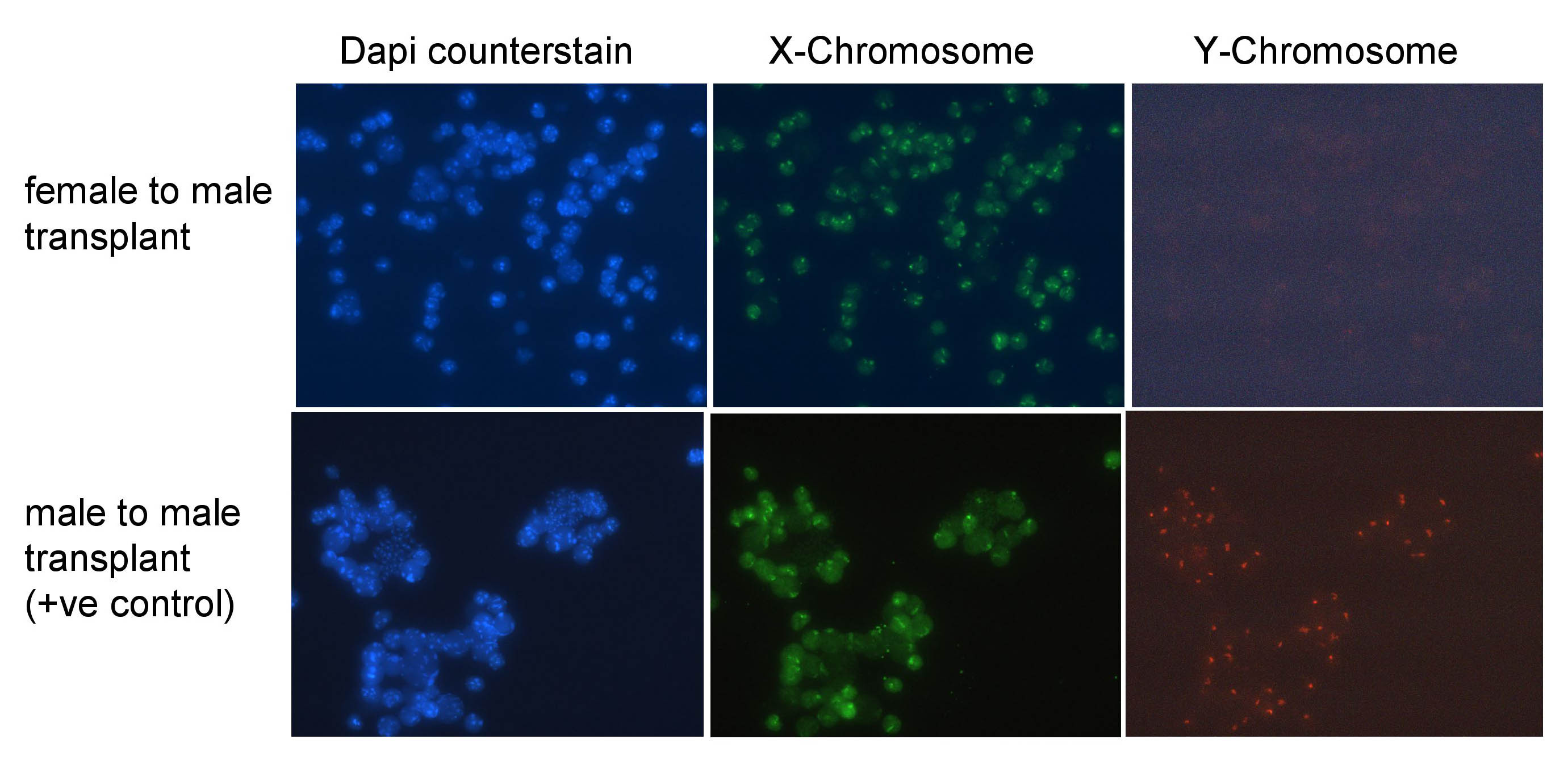

Supplement: Figure S3 — Chromosome painting of bone-marrow transplanted mice. No Y-chromosomes were seen in male mice transplanted with female bone marrow, confirming engraftment was successful. Male recipients of male bone marrow all have Y-chromosomes, as expected, as a positive control for this method. (2.45 MB TIF) [file pone.0005073.s005.tif]

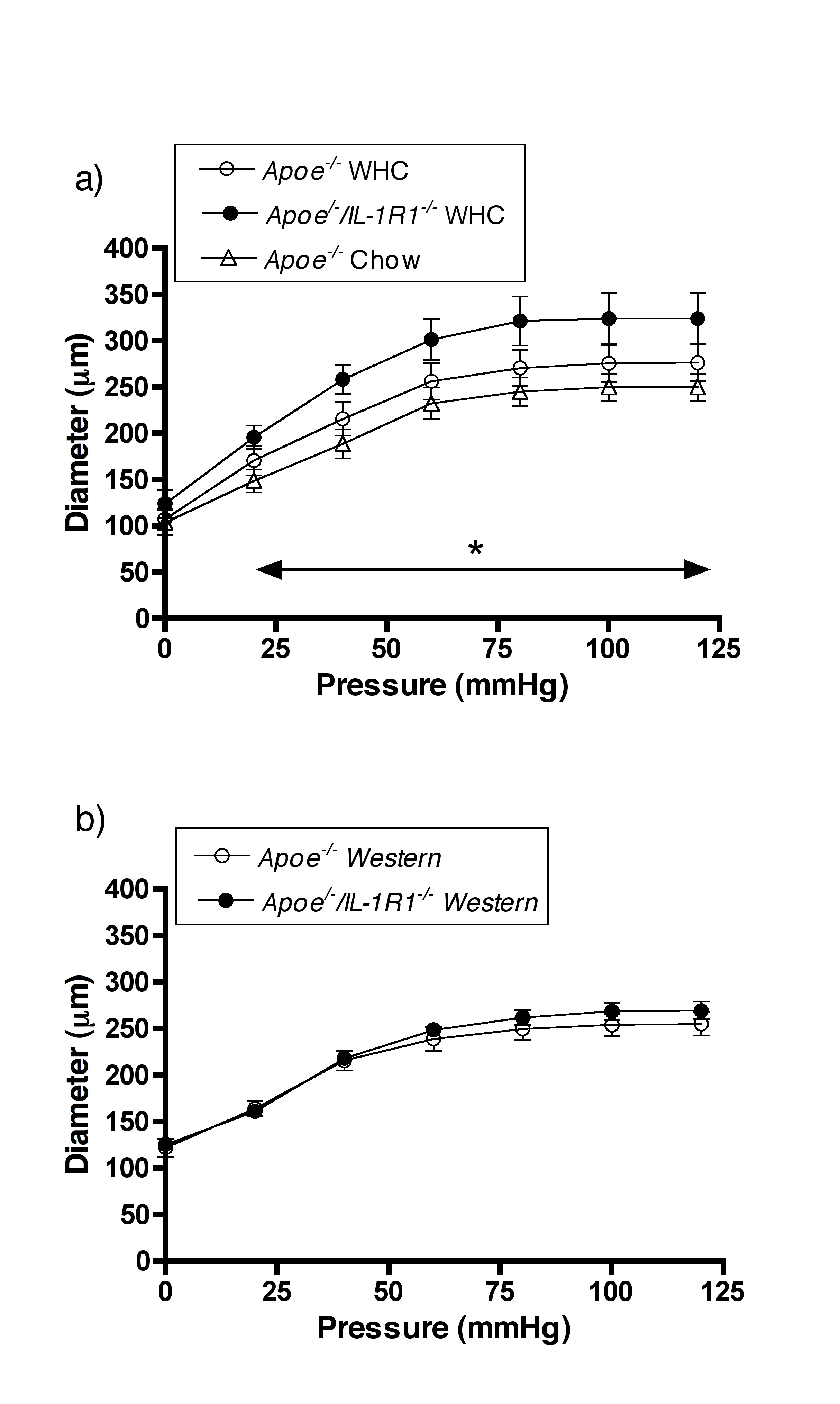

Supplement: Figure S4 — Vascular reactivity of arterioles from Apoe−/− and Apoe−/−/IL-R1−/− mice. Intraluminal arteriolar diameter in response to increasing pressure (0–120 mmHg). *P<0.05 Apoe−/−/IL-R1−/− (n = 6) and **P<0.05 Apoe−/− WHC (n = 6) versus Apoe−/− chow (n = 4). (0.61 MB TIF) [file pone.0005073.s006.tif]

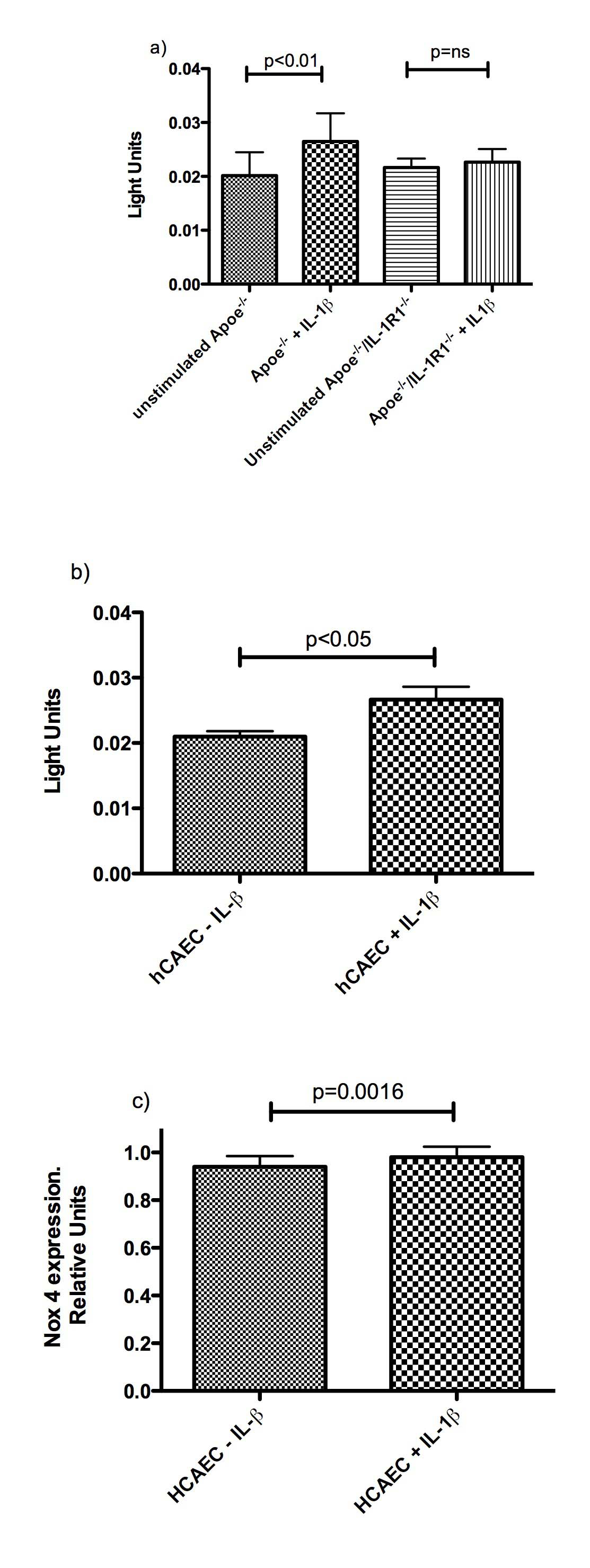

Supplement: Figure S5 — ROS and Nox 4 expression in endothelial cells in culture. Increased ROS are seen following IL-1β stimulation of endothelial cells isolated from Apoe−/− mice (n = 6) (a) and human coronary endothelial cells (hCAEC) (n = 6) (b). However, no increase in ROS are seen in ECs isolated from Apoe−/−/IL-1R1−/− mice (n = 6) (a). Nox 4 mRNA expression is increased in hCAEC stimulated with IL-1β (n = 5) (c). (9.90 MB TIF) [file pone.0005073.s007.tif]

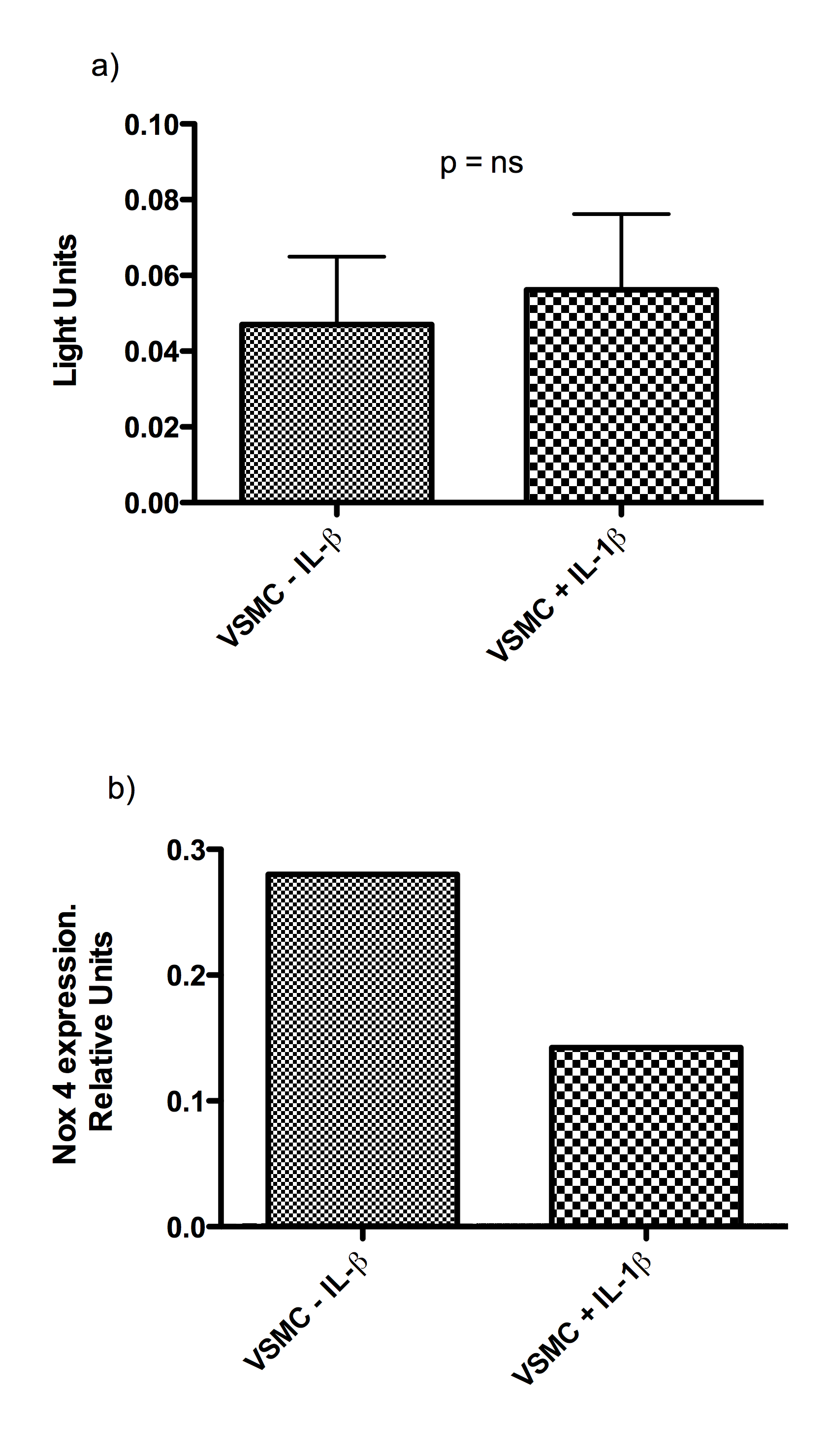

Supplement: Figure S6 — ROS and Nox 4 expression in vascular smooth muscle cells in culture. No significant difference in ROS are seen following IL-1β stimulation of VSMCs (n = 6) (a). Nox 4 mRNA expression is decreased in VSMC stimulated with IL-1β (n = 1) (b). (0.30 MB TIF) [file pone.0005073.s008.tif]

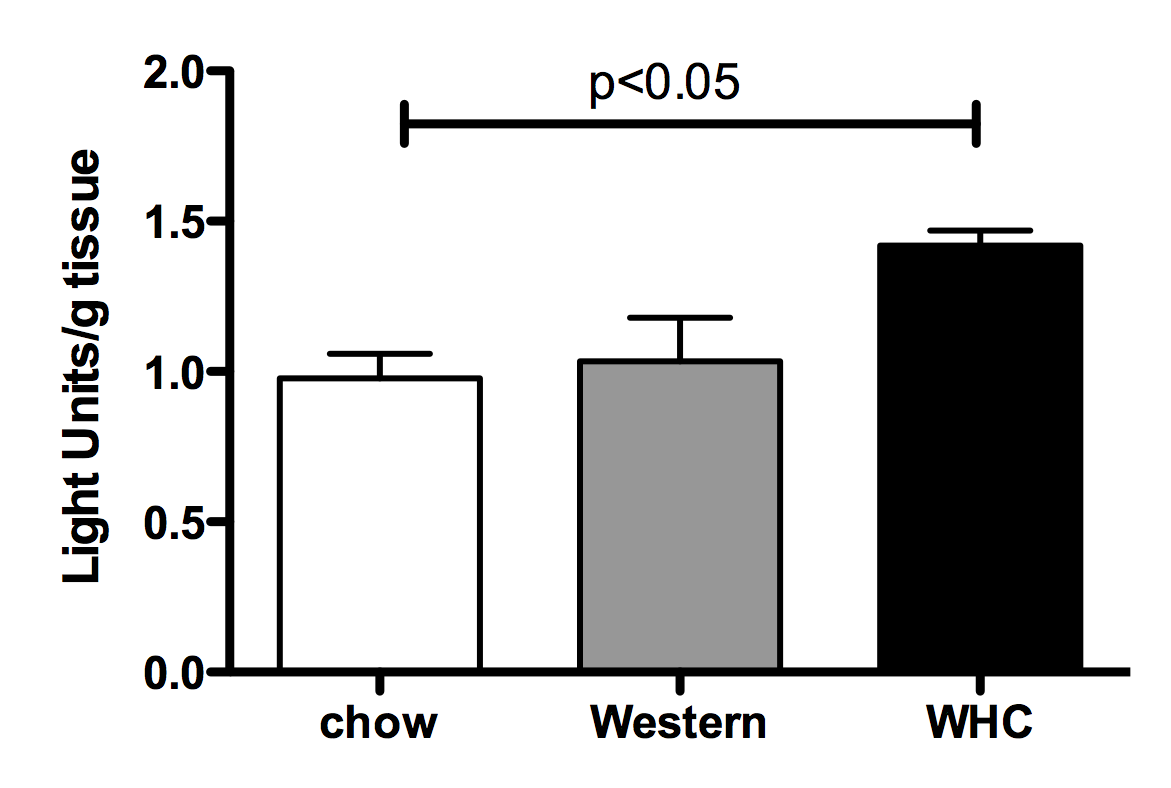

Supplement: Figure S7 — ROS generation in Apoe−/− mice following feeding of a high fat diet. Mice fed WHC had significantly more ROS than those fed chow alone, with Western diet giving intermediate levels of ROS. (n = 9). (2.79 MB TIF) [file pone.0005073.s009.tif]
