# Supplementary material for: Interleukin-1 Regulates Multiple Atherogenic Mechanisms in Response to Fat Feeding
Source: PLoS One. 2009 Apr 6;4(4):e5073. doi: 10.1371/journal.pone.0005073 (PMC2661361; doi:10.1371/journal.pone.0005073)
Supplement: Table S1 — Lipid, glucose and ALT levels in ApoE−/−/IL-1R1−/− and ApoE−/− mice fed chow, Western High Cholate (WHC), and Western diets. Data represents mean+/−SEM. (0.05 MB DOC) [file pone.0005073.s010.doc]

**Table S1:** Lipid, glucose and ALT levels in ApoE^-/-^/IL-1R1^-/-^ and ApoE^-/-^ mice fed chow, Western High Cholate (WHC), and Western diets. Data represents mean +/- SEM.

|  | ApoE^-/-^ | | | ApoE^-/-^/IL-1R1^-/-^ | | |
| --- | --- | --- | --- | --- | --- | --- |
|  | Chow | Western | WHC | Chow | Western | WHC |
| Tryglyceride (mmol) | 1.1+/-0.2 | 1.85+/0.27 | 3.72+/-0.35* | 1.3+/-0.4 | 3.38+/-0.46 | 3.28+/-0.55 |
| Cholesterol (mmol/L) | 17.4+/-2.8 | 19.77+/-1.13 | 39.12+/-2.63† | 10.9+/-2.63 | 22.37+/-1.53 | 34.48+/-2.35‡ |
| HDL (IU/L) | 3.52+/-0.58 | 3.61+/-0.3 | 8.2+/-0.39† | 1.87+/-0.07 | 3.74+/-0.24 | 7.75+/-0.57§ |
| Cholesterol:HDL | 4.95+/-0.05 | 5.62+/-0.46 | 4.7+/-0.2 | 5.8+/-0.1 | 6.0+/-0.28 | 4.64+/-0.04 |
| Glucose (mmol/L) | 12.1+/-1.6 | 11.13+/-0.61 | 8.5+/-0.64 | 8.55+/-0.45 | 12.4+/-0.71 | 9.25+/-1.49 |
| ALT (IU/L) | 30.0+/-5.0 | 40.67+/-12.55 | 93.83+/-35.73 | 107.0+/-72.0 | 142.0+/-32.7 | 146.3+/-33.49 |

* p<0.05 vs ApoE^-/-^ chow and ApoE^-/-^ Western

† p<0.001 vs ApoE^-/-^ chow and ApoE^-/-^ Western

‡ p<0.001 vs ApoE^-/-^/IL-1R1^-/-^ chow, p<0.01 vs ApoE^-/-^/IL-1R1^-/-^ Western

§ p<0.001 vs ApoE^-/-^/IL-1R1^-/-^ chow, and ApoE^-/-^/IL-1R1^-/-^ Western
