# Supplementary material for: Interleukin-1 Regulates Multiple Atherogenic Mechanisms in Response to Fat Feeding
Source: PLoS One. 2009 Apr 6;4(4):e5073. doi: 10.1371/journal.pone.0005073 (PMC2661361; doi:10.1371/journal.pone.0005073)
Supplement: Table S2 — Plasma levels of IL-1ra, IL-1β, IL-1α and IL-6 in ApoE−/−/IL-1R1−/− and ApoE−/− mice fed chow, Western high cholate (WHC), and Western diets, determined by ELISA. Data represents mean+/−SEM. (0.04 MB DOC) [file pone.0005073.s011.doc]

Table S2: Plasma levels of IL-1ra, IL-1 IL-1α and IL-6 in ApoE^-/-^/IL-1R1^-/-^ and ApoE^-/-^ mice fed chow, Western high cholate (WHC), and Western diets, determined by ELISA. Data represents mean +/- SEM.

|  | ApoE^-/-^ | | | ApoE^-/-^/IL-1R1^-/-^ | | |
| --- | --- | --- | --- | --- | --- | --- |
|  | Chow | Western | WHC | Chow | Western | WHC |
| IL-1ra (pg/ml) | 14.5+/-0.98 | 15.79+/-2.17 | 29.51+/-14.46 | 23.98+/-1.16 | 55.92+/-38.14 | 93.38+/-52.98 |
| IL-1β (pg/ml) | 8.14+/-1.9 | 12.06+/-1.17 | 7.88+/-0.61 | 5.64+/-0.20 | 8.22+/-0.48 | 12.07+/-2.71 |
| IL-1α (pg/ml) | 21.6+/-6.99 | 32.0+/-4.13 | 22.84+/-3.95 | 37.53+/-8.29 | 37.48+/-9.07 | 37.04+/-2.31 |
| IL-6 (pg/ml) | 5.23+/-0.50 | 12.91+/-1.91 | 8.18+/-0.59 | 3.14+/-1.06 | 4.32+/-0.30* | 9.64+/-1.50† |

* p<0.001 vs ApoE^-/-^ Western

† p<0.05 vs ApoE^-/-^/IL-1R1^-/-^ Western
