# Supplementary material for: Interleukin-1 Regulates Multiple Atherogenic Mechanisms in Response to Fat Feeding
Source: PLoS One. 2009 Apr 6;4(4):e5073. doi: 10.1371/journal.pone.0005073 (PMC2661361; doi:10.1371/journal.pone.0005073)
Supplement: Table S3 — Potency and efficacy of vasoconstrictor response to phenylephrine in pressurised mesenteric arterioles from Apoe−/−/IL1R1−/− and Apoe−/− mice fed a Western high cholate (WHC), Western, or Apoe−/− mice fed chow diet, assessed by pD2 (negative logarithm of EC50: concentration required for half maximum response) and Emax (percentage maximum constriction). All data are means+SEM. p = ns. (0.04 MB DOC) [file pone.0005073.s012.doc]

**Table S3: Potency and efficacy of vasoconstrictor response to phenylephrine in pressurised mesenteric arterioles from *Apo e-/-/IL1R1-/-* and *Apo e-/-* mice fed a Western high cholate (WHC), Western, or *Apo e-/-* mice fed chow diet, assessed by pD_2_ (negative logarithm of EC_50_: concentration required for half maximum response) and E_max_ (percentage maximum constriction). All data are means +SEM. p=ns.**

|  | ApoE^-/-^ | | | ApoE^-/-^/IL-1R1^-/-^ | |
| --- | --- | --- | --- | --- | --- |
|  | Chow (n=4) | Western (n=5) | WHC (n=6) | Western (n=5) | WHC (n=6) |
| PD_2_ | 6.2+/-0.2 | 6.3+/-0.1 | 6.5+/-0.1 | 5.6+/-0.5 | 6.6+/-0.1 |
| E_max_ | 75.5+/-3.1 | 66.8+/-4.1 | 75.2+/-5.4 | 74.7+/-5.4 | 73.6+/-1.7 |
